# Boron Nitride Doped Polyhydroxyalkanoate/Chitosan Nanocomposite for Antibacterial and Biological Applications

**DOI:** 10.3390/nano9040645

**Published:** 2019-04-21

**Authors:** Abdul Mukheem, Syed Shahabuddin, Noor Akbar, Azizi Miskon, Norazilawati Muhamad Sarih, Kumar Sudesh, Naveed Ahmed Khan, Rahman Saidur, Nanthini Sridewi

**Affiliations:** 1 Department of Maritime Science and Technology, Faculty of Defence Science and Technology, National Defence University of Malaysia, Kuala Lumpur 57000, Malaysia; azizimis@gmail.com; 2 Research Centre for Nano-Materials and Energy Technology (RCNMET), School of Science and Technology, Sunway University, Subang Jaya 47500, Malaysia; saidur@sunway.edu.my; 3 Department of Biological Sciences, School of Science and Technology, Sunway University, Subang Jaya 47500, Malaysia; noormicrobiologist555@gmail.com (N.A.); naveedk@sunway.edu.my (N.A.K.); 4 Polymer Research Laboratory, Department of Chemistry, University of Malaya, Faculty of Science, Kuala Lumpur 50603, Malaysia; nmsarih@um.edu.my; 5 Applied Microbiology and Ecobiomaterial Research Laboratory, School of Biological Sciences, Universiti Sains Malaysia, Penang 11800, Malaysia; ksudesh@usm.my; 6 Department of Engineering, Lancaster University, Lancaster LA1 4YW, UK

**Keywords:** boron nitride, chitosan, polyhydroxyalkanoate, nanocomposite, biocompatible, antibacterial

## Abstract

The present research focused on the fabrication of biocompatible polyhydroxyalkanoate, chitosan, and hexagonal boron nitride incorporated (PHA/Ch-hBN) nanocomposites through a simple solvent casting technique. The fabricated nanocomposites were comprehensively characterized by Fourier transform infrared spectroscope (FT-IR), field emission scanning electroscope (FESEM), and elemental mapping and thermogravimetric analysis (TGA). The antibacterial activity of nanocomposites were investigated through time-kill method against multi drug resistant (MDR) microbes such as methicillin-resistant *Staphylococcus aureus* (MRSA) and *Escherichia coli* (*E. coli*) K1 strains. In addition, nanocomposites have examined for their host cytotoxicity abilities using a Lactate dehydrogenase (LDH) assay against spontaneously immortalized human keratinocytes (HaCaT) cell lines. The results demonstrated highly significant antibacterial activity against MDR organisms and also significant cell viability as compared to the positive control. The fabricated PHA/Ch-hBN nanocomposite demonstrated effective antimicrobial and biocompatibility properties that would feasibly suit antibacterial and biomedical applications.

## 1. Introduction

In recent years, with the rapid development in biomedical field, many diseases have been discovered, and many have evolved due to various changes in the biological environment. Microbial infections are the key source of chronic diseases and mortality. There are few multi-drug resistant microbes, namely *E. coli* and *S. aureus*, which are generally responsible for infections such as wound in soft tissues, skin, and bloodstream diseases [1,2]. Antibiotics are the ideal treatment for bacterial infections because of their broad-spectrum effective outcomes [3]. However, many studies have revealed that the extensive use and abuse of antibiotics has led to the emergence of multidrug resistant microbes, which is a major weakness of current antibiotic treatment [4]. Besides, cytocompatibility remains one of the most important features which has to be accounted for while proposing any antibiotic technique. Thus, the present scenario motivated scientists to develop biocompatible nanocomposites that feasibly exert better antibacterial and biocompatible properties including cost effectiveness.

Nanomaterials have multiple modes of action within microbial cells that lead to their death, all of which have been rays of hope for providing minimal death resistance compare to conventional antibiotics and anticancer drugs [5]. Many nanomaterials, for example graphene, silver, and gold, have demonstrated excellent antimicrobial properties which can be exploited in various biomedical applications [6,7]. Boron nitride (BN) is a strong carbon material formed by the bonding of boron and nitrogen elements. Furthermore, hBN has been extensively explored in biomedical applications and reported as biocompatible material in various biological applications [8,9]. In a study, an optimal viability of PC12 cells was noticed up to a 50 µg mL^−1^ concentration of hBN, and there was no formation reactive oxygen species (ROS), cellular changes, or apoptosis for up to nine days of the experiment [10]. Biodegradable nanocomposites have also been designed by reinforcing hBN nanotubes, which have demonstrated significant enhancement in properties such as time, durability of scaffold, non-toxicity on osteoblasts and macrophages, and improved cell proliferation, which are apposite requirements for biomedical applications [11,12]. Merih et al. reported antibacterial and antibiofilm characteristics of hBN nanoparticles. The research revealed that *Streptococcus mutans* and *Staphylococcus pasteuri* obtained higher minimal inhibitory concentration value compared to *Streptococcus mutans* 3.3 and *Candida* species. [13]. Typically, *Streptococcus mutans* was known as an early resident that colonizes the tooth surface, forming dental plaque and the main pathogens for dental caries [13]. Thus, for the aforementioned potential of hBN, it must be further investigated for its broad spectrum antibacterial and host cytotoxicity effects, respectively. 

Currently bio-based polymers are of great interest in biomedical research due to their inherent characteristics [14,15,16]. Biopolymers have been explored to achieve systemic improved therapeutics such as sustained drug release, even drug distribution, and set period degradation [17]. Polyhydroxyalkanoate is the class of novel biopolymers which belongs to the family of natural polyesters containing many different hydroxyl and carboxylic functional groups [18]. Furthermore, PHA is produced by several Gram-negative and Gram-positive microbes when fed with excess quantity of carbon under stressed growth conditions [19]. This unfavorable condition forced the microbe to store energy and carbon in the form of PHA macromolecules [19]. PHA has attracted a considerable scientific attention due to its biocompatible nature and biodegradability properties in a variety of applications ranging from nanotechnology, medical, tissue engineering, and packing industries [20,21,22,23]. However, PHA possesses limited applications in biomedical field due to its dissolution in toxic solvents such as chloroform, large crystals that cause brittleness and poor mechanical properties that are not suitable for biomaterials, wound management and packing [24,25]. Therefore, to improve the mechanical and thermal properties of PHA, its monomer side chain plays vital role and can be copolymerized with different monomers such as 3-hydroxyhexanoate (HHx), providing better flexibility, biodegradability, and mechanical properties, compared to bare PHA [24,26]. 

Furthermore, blending is one of the most suitable and easy methods of improving the incompatible properties of PHA by mixing with other biopolymers, a method which is hypothesized to have excellent physiochemical properties [24]. Chitosan is a natural cationic polysaccharide which has generated considerable interest due to its unique properties, and it is commonly used for the modification of drug formulations [27,28]. Saeed et al. reported that a Polyhydroxybutyrate chiotsan (PHB/Ch) matrix has demonstrated a more improved degradation rate than the individual efficacies of PHB. They found that dissolution of chitosan feasibly neutralizes the acidic nature of PHB degradation products [29]. Thus, chitosan’s cross-linking ability helps to blend with various biocompatible polymers and nanoparticles in order to feasibly enhance composite properties such as surface morphology, contact angle length, and degradability [28].

The purpose of the present research is to evaluate the potential antibacterial effect and cell viability efficacy of fabricated PHA/Ch-hBN nanocomposites that were loaded with three different concentrations of hBN nanoparticles. Therefore, to achieve this purpose, the antibacterial activity of nanocomposites were analyzed against multi-drug resistant *E. coli* K1 and methicillin-resistant *Staphylococcus aureus* (MRSA). In addition, the cell cytotoxicity assays have also been examined through the LDH method. Additionally, in the present study nontoxic acetic acid was used to dissolve the PHA instead of a standard solvent chloroform to dissolve PHA [30].

## 2. Materials and Methods

Poly 3-hydroxybuterate-co-12mol% hydroxyhexanoate (P3HB-co-12mol%HHx) powder (350,000 Da) was provided by the KANEKA corporation, Osaka Japan. Chitosan powder of a medium molecular weight was obtained from Sigma-Aldrich, St. Louis, MO, USA. Hexagonal Boron Nitride (hBN) powder with an average particle size of 70 nm was procured from Lower Friction Company (Ontario, Canada). All the analytical grade reagents were used throughout the experiment. For antibacterial assays, stationary phase bacterial strains of methicillin-resistant *Staphylococcus aureus* (MRSA) and *E. coli* (K1) were used. Moreover, spontaneously immortalized human keratinocytes (HaCaT) cell lines were used for lactate dehydrogenase assay. A cytotoxicity detection kit was purchased from Roche Diagnostics, Indianapolis, IN, USA.

### 2.1. Preparation of Precursor Solution

A polymer solution with a concentration of 1 mg/mL of PHA in glacial acetic acid was prepared using a standard reflux setup. In a round bottom flask, PHA powder was mixed with glacial acetic acid and stirred for 5 min (300 rpm) at 118 °C on a magnetic stirrer. The solution was then allowed to cool down at room temperature. Simultaneously, a chitosan solution with a concentration of 1 mg/mL was prepared in 1% acetic acid, which was stirred for 24 h at room temperature on a magnetic stirrer. Furthermore, hBN nanoparticles with different weight percentages with respect to PHA (0.1, 0.5, and 1) were mixed in PHA solutions using a magnetic stirrer followed by ultra-sonication to dispersed the nanoparticles. The prepared chitosan solution (1 mg/mL) was added dropwise into the hBN-doped PHA solution to obtain a 10:1 ratio of PHA/Ch. The resultant PHA/Ch-hBN solution was ultra-sonicated for 30 min to distribute nanoparticles evenly within the polymer matrix. In this experiment, three nanocomposites were prepared with different concentrations of hBN nanoparticles.

### 2.2. Solvent Casting

**Figure 1:**
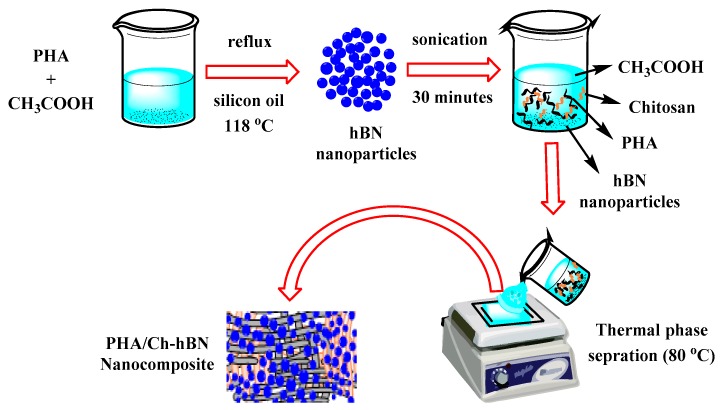
Schematic representation of the fabrication of film via solvent casting technique.

A nanocomposite film was prepared from the polymer solution as illustrated in Figure 1. The solvent casting stage was prepared using a flat glass plate while the film size and thickness were controlled by standard microbiological glass slides (75 mm × 25 mm × 1 mm). A polymer solution was dispensed carefully onto the preheated (80 °C) glass platform with complete evaporation of solvent leading to the formation of uniformly thin film. PHA/Ch and PHA films were also prepared using a similar method. The resultant nanocomposites were dried at room temperature to remove any further residuals solvent.

### 2.3. Antibacterial Assays

Antibacterial assays were performed to determine the percent bactericidal activity of different nanocomposites against multi-drug resistant bacteria. Briefly, 1 × 10^6^ bacterial cells were incubated with PHA, PHA/Ch and PHA/Ch-hBN nanocomposites at 37 °C for 0, 2, 4, 6, and 24 h using the time-kill method. This method could be appropriate for the interaction between antibacterial agent and cultural broth. The clinical and laboratory standards institute (CLSI) has described this method as a well standardized technique to use for bacteria that is documented in M26-A of CLSI [31]. Bacterial cultures of MDR *E. coli* K1 and MRSA were grown over night, and the density of c. 1 × 10^8^ CFU/mL was obtained through measuring 0.22 optical density (OD) at 595 nm [32]. About 10 µL, which is equal to c. 1 × 10^6^ CFU/mL, was added in 100 µL nutrient broth containing the test samples of size 0.6 mm. The phosphate buffer saline was used to mark the total volume up to 200 µL. Next, cultures were ten-fold serially diluted to 10 µL, were plated on nutrient agar plates, and were incubated over night at 37 °C. For controls, bacteria incubated with gentamicin (100 µg/mL) as a positive, and PBS and PHA including PHA/Ch were used as negative control. The experiment was performed and analysed in triplicate.

### 2.4. Cytotoxicity Assays

**Figure 2:**
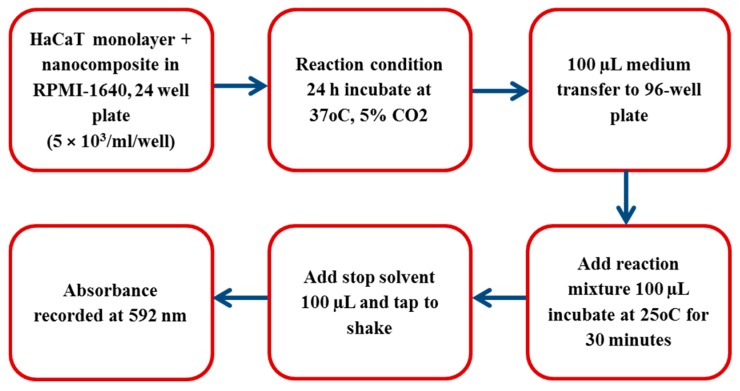
Schematic representation of measuring lactate dehydrogenase.

HaCaT cell lines were grown in RPMI-1640 medium and supplemented with 10% (v/v) foetal bovine serum (FBS), 1% L-glutamine, 1% Penicillin-Streptomycin, and 1% minimal essential media nonessential amino acid (Life Technologies, California, CA, USA). The HaCaT cells were been seeded into the non-pyrogenic 24 well plate with the density of 5 × 10^3^ per well and incubated at 37 °C with 5% CO_2_, including 95% humidity, for 48 h. Briefly, a HaCaT cells monolayer was incubated with nanocomposites in a RPMI-1640 medium for 24 h at 37 °C in the presence of 5% CO_2_ and 95% humidity. After this incubation, supernatant was aspirated to detect the cytotoxicity through measuring the release of lactate dehydrogenase (LDH), a schematic presentation of which is shown in Figure 2. Generally, the results of cytotoxicity were calculated applying the below formula:

$$\%\;\mathrm{cytotoxicity}=\frac{\mathrm{sample}\;\mathrm{value}-\mathrm{control}\;\mathrm{value}}{\mathrm{total}\;\mathrm{LDH}\;\mathrm{release}-\mathrm{control}\;\mathrm{value}\;}\times 100$$

Whereas, to obtained the total LDH release, HaCaT cells were treated with 0.1% Triton X-100 at 37 °C for 30 min. Similarly, the cells incubated in RPMI-1640 were used as control values. In brief, the working principle of the assay was that LDH acted as catalyst for transforming lactate to pyruvate, thereby producing NADH and H^+^. In next step, catalyst diaphorase converted H and H^+^ to tetrazolium salt that was then reduced to formazan dye. The relationship between metabolically active cells and formazan dye color provided precise quantification (cell death or proliferation) from the resultant supernatant. A dense purple color feasibly demonstrated the high enzyme activity due to the higher viable cells, and decrease in purple color signified the cytotoxicity.

## 3. Results

### 3.1. Morphological Analysis

**Figure 3:**
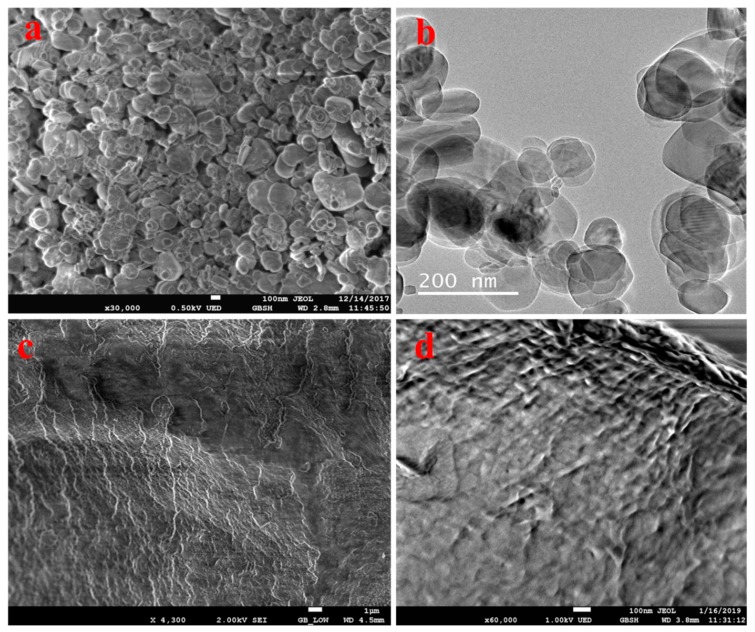
FESEM images of hBN (**a**), chitosan (**c**), and a PHA/Ch-hBN nanocomposite (**d**), as well as a TEM image of hBN (**b**).

A surface morphological analysis of the hBN nanosheets, chitosan, and PHA/Ch-hBN nanocomposites were comprehensively evaluated through FESEM and TEM, as shown in Figure 3. Figure 3a represents the FESEM image of hBN which has depicted the discs like morphology in nano range where the 2D hBN flakes can be seen densely stacked onto each other. In addition, the obtained TEM image further confirm the disc-like morphology of hBN nanoflakes (Figure 3b). The stacked disc-like morphology of hBN, a characteristic of 2D, has been clearly established by FESEM and TEM analysis. The FESEM micrograph of chitosan is shown in Figure 3c, which demonstrates the cotton flakes like structures that is the characteristic morphology of chitosan [33]. Similar topology was also observed in nanocomposite depicted in Figure 3d, which is clearly revealing the formation of cross-linked polymeric networking of PHA/Ch. The morphology of the nanocomposites appears to be 2D layered type, which might be due to the interaction of polymeric chains with 2D hBN. However, from Figure 3d, it was found that the hBN nanoflakes were difficult to visualize within the nanocomposite since hBN nanodiscs were deeply embedded in the PHA polymeric matrix and difficult to visualize using FESEM imaging alone. Additionally, the amount of hBN is much less as compared to the bare polymer matrix, hence making its analysis further cumbersome through FESEM single-handedly. Therefore, the SEM elemental mapping analysis technique appears to be an appropriate method to demonstrate the presence and even distribution of hBN within the PHA/Ch matrix; this was further supported by energy dispersive X-ray EDX investigations. 

**Figure 4:**
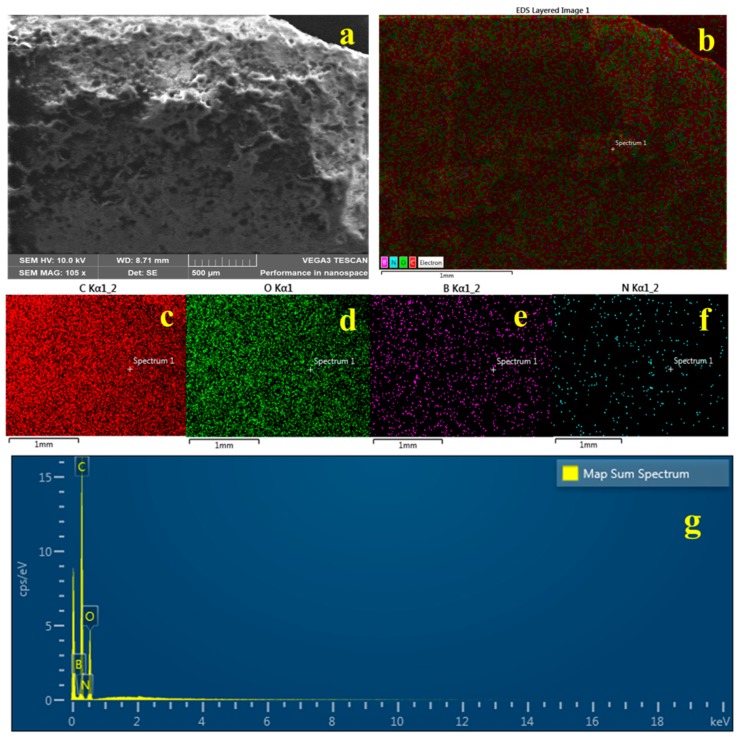
SEM image (**a**), EDX (**g**) and mapping (**b**–**f**) of PHA/Ch blend doped with 0.1% hBN nanocomposites.

Figure 4a,b illustrates the elemental mapping results of the composite PHA/Ch-hBN-5. As obvious from the obtained elemental mapping outcomes in Figure 4, hBN (Figure 4e,f) is present uniformly and homogeneously within the nanocomposites in addition with carbon (c) and oxygen (d), thereby confirming the successful formation of 2D hBN doped PHA/Ch nanocomposite. The elemental analysis was further found to be in a good agreement with the EDX data presented in Figure 4g, which clearly reveals the presence of B and N within the nanocomposite scaffold.

### 3.2. FT-IR

**Figure 5:**
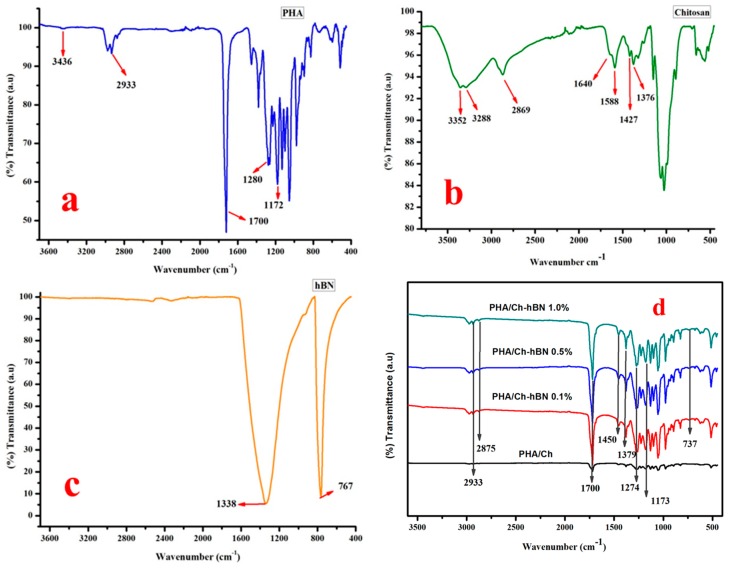
FT-IR (Fourier-transform infrared spectroscopy) spectrum of bare (**a**) PHA, (**b**) Chitosan, (**c**) hBN and, (**d**) nanocomposites.

The FT-IR spectra of PHA, Chitosan, hBN, PHA/Ch and PHA/Ch doped hBN nanocomposites are shown in Figure 5. The FT-IR spectrum of PHA is illustrated in Figure 5a. The IR peak appearing at 1700 cm^−1^ may be assigned to C=O (ester carbonyl) stretching of PHA polymer. The prominent bands at the spectral region of 1172 to 1280 cm^−1^ are associated C-O-C vibrations within the PHA polymeric chains. The IR bands appearing around at 3436 cm^−1^ can be assigned to -OH vibration in the carboxyl group present in copolymeric chains of PHA and P(3HB-co-3HHx). The prominent IR peak at 2933 cm^−1^ is the characteristic -C-H- vibrations of the methylene group present in the macromolecules of PHA (Figure 5a) [34,35]. Figure 5b exemplifies the FT-IR spectrum of chitosan. The broad IR bands at the range of 3352–3288 cm^−1^ may be assigned to stretching modes of the –OH and primary amine groups. The characteristic IR peaks appearing at 1640 cm^−1^ and 1588 cm^−1^ might be attributed to the C-N and C=O (amide) stretching modes. The C-H stretching and bending was ascribed to the bands at the spectral region of 2869 cm^−1^ and 1427 cm^−1^, respectively (Figure 5b) [36]. From the hBN spectra shown in Figure 5c, the typical stretching vibration bands of B-N bond and B-N-B bending vibration were attributed around the spectral region of 1338 cm^−1^ and 767 cm^−1^ [37,38]. The IR spectrum of the various nanocomposites scaffolds are represented in Figure 5d. As obvious from the spectra of the nanocomposites, they comprise of the characteristic peaks and IR bands of PHA, chitosan, and hBN. As a result of an overlapping of the C-H stretching of chitosan in composites, the IR band at approximately 2877 cm^−1^ became less intense and was been slightly shifted to 2875 cm^−1^. In addition, the C=O (amide) and C-N stretching bands in chitosan around at 1640 cm^−1^ and 1588 cm^−1^, respectively, vanished in composites. The absorption bands at 2930 cm^−1^ and 1450 cm^−1^ in bare PHA and chitosan showed slight shifts in the composites. The characteristic peaks at 1338 cm^−1^ and 767 cm^−1^ in hBN were detected at 1379 cm^−1^ and 738 cm^−1^ in hBN doped PHA/Ch nanocomposites. However, many IR peaks and bands have depicted significant changes shift as compared to the spectrum of of bare PHA, chitosan, and hBN signifying, the chemical associations or interactions with each other. Thus, the FT-IR spectrum suggests the formation of a PHA/Ch-hBN nanocomposite, which is in good agreement with the earlier analysis. 

### 3.3. Thermal Gravimetric Analysis (TGA)

**Figure 6:**
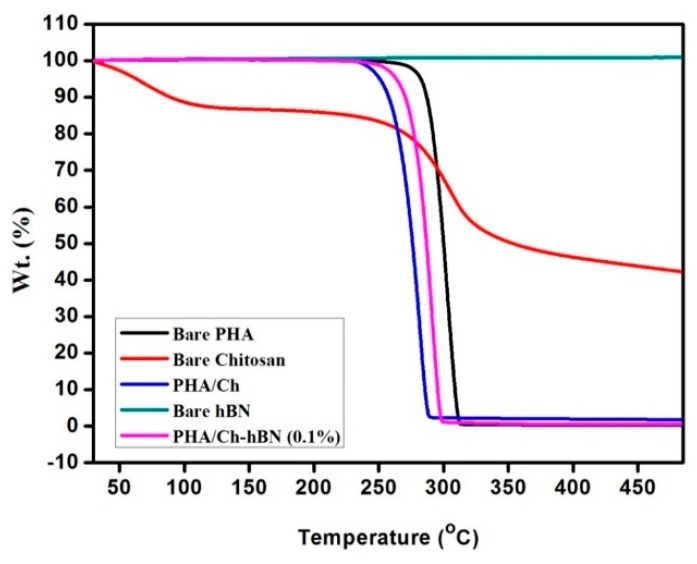
TGA thermogram of bare PHA, chitosan, hBN, and PHA/Ch, PHA/Ch-hBN nanocomposites.

Figure 6 represented the TGA results of PHA/Ch and PHA/Ch-hBN nanocomposites including bare PHA, chitosan, and hBN. The TGA analysis was performed under a nitrogen atmosphere at a temperature range of 30–500 °C for 10 °C/min. As apparent from the TGA thermogram of hBN, no weight loss pccured until 500 °C, which indicated a higher level of thermal stability. On the other hand, chitosan has revealed a 10% weight loss between 50–115 °C, which can be attributed to the loss of residual water and adsorbed moisture. The major weight loss in chitosan is observed at 250–343 °C, which may be assigned to the decomposition of ethereal groups, glucosamine moieties, and ring opening reactions. The thermogram of PHA reveals that it is thermally stable until 200 °C, the gradual weight loss has initiated around 250 °C, and a considerable weight loss of 98.6% was noted form 268–310 °C. This thermal degradation of PHA may be due to the degradation of unreacted free monomers and the decomposition of hydrocarbon polymeric chains. The blend of PHA/Ch has shown less thermal stability as bare compared to PHA but higher stability as compared to chitosan, which indicates some interactions and crosslinking between the two polymers. Though PHA has shown 100% decomposition till 500 °C, PHA/Ch has revealed 4% residue at 500 °C, thus signifying some synergistic associations between tow biopolymers imparting thermal stability. Finally, the PHA/Ch-hBN nanocomposite revealed the improved thermal stability compared to PHA/Ch composite, which is possibly due to the addition more thermally stable hBN nanomaterial. Therefore, from the analysis, it was shown that the thermal stability of PHA/Ch-hBN nanocomposite has been improved as compared to PHA/Ch and can be applied in applications which require higher temperature (Up to 250 °C). 

## 4. Antibacterial Analysis

**Figure 7:**
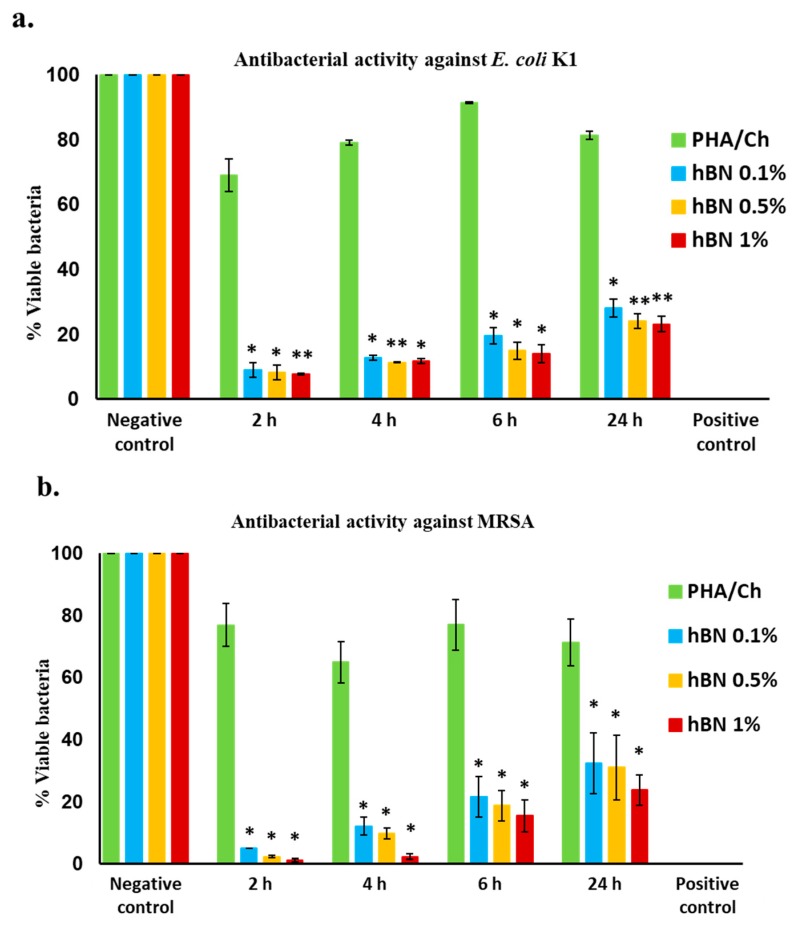
(**a**) Bactericidal activity of nanocomposites against *E. coli* K1 strain and (**b**) bactericidal activity against MRSA, shown significant bactericidal efficacy. (Using a two sample T-test, two-tailed distribution, (*) is <0.05; (**) is <0.01; and (***) is <0.001).

The antibacterial activities of fabricated nanocomposites were tested against MDR *E. coli* K1 and MRSA through the time-kill method. The results of PHA/Ch-hBN (hBN 0.1, 0.5 and 1.0 wt% wrt PHA) exhibited significant bactericidal activity against *E. coli* K1 (*p* < 0.05), which is represented in Figure 7a and Table 1. When tested against MRSA, the results revealed that nanocomposites significantly reduced the growth of bacteria (*p*-value) (<0.05) (Figure 7b and Table 1). The hBN nanocomposites remarkably reduce the percent viability of bacteria as compared to negative control (Figure 7a,b). 

In brief, the antibacterial activity was assessed at different time intervals, and the first 2, 4, and 6 h of time points demonstrated effective bactericidal activity as compared to negative control (PHA/Ch), including 24 hours’ time point. From the graph, it is hypothesized that the bactericidal activity was time dependent in this experiment, which was subjected to the morphology of nanocomposites. Furthermore, an increase in the concentration of hBN nanomaterial with the PHA/Ch composition has revealed the improved bactericidal ability. However, all the three concentrations of hBN nanocomposites have shown a significant reduction of *E. coli* K1 and MRSA for up to 24 h. The Gentamicin was applied as positive control, which killed 100% of the bacterial colonies. The statistical *p*-values were defined by applying two sample T-test distribution, while (*) is <0.05; (**) is <0.01; and (***) is <0.001, respectively.

## 5. Cell Cytotoxicity Assay against HaCaT Cell Lines

**Figure 8:**
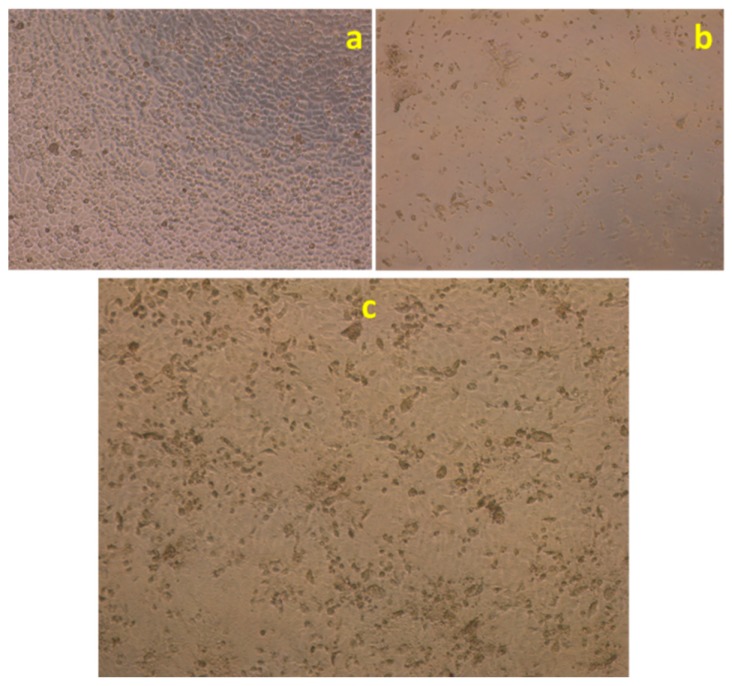
Effects of synthesized nanocomposites on the viability of HaCaT cell line, (**a**–**c**) represent the negative controls, positive controls, and PHA/Ch-hBN nanocomposite, respectively.

For the biomedical applications, cell viability testing is an important step to determine the cellular effects of external toxicants and the biosafety of biomaterials. Therefore, the synthesized PHA/Ch-hBN nanocomposites were tested for their cytotoxic effects against HaCaT cell lines, and the cell viability was quantified through LDH assays. HaCaT cells have treated with test samples for up to 24 h, and the resultant cells have visualized by inverted microscope at 200X lens, which is presented in Figure 8. 

**Figure 9:**
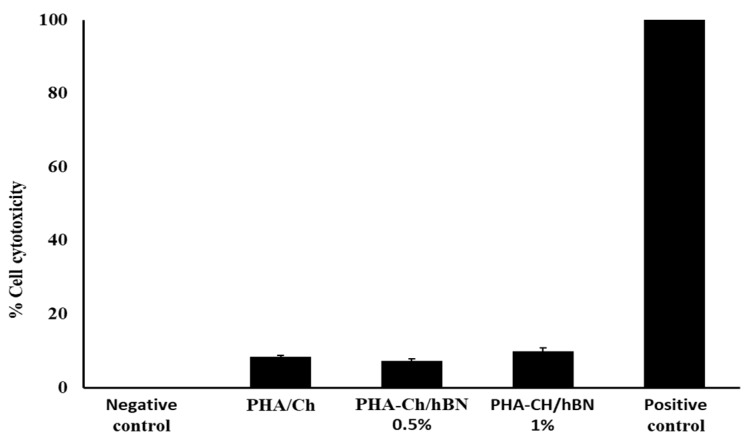
Representative cytotoxic effects of synthesized nanocomposites and positive control (1% Trixton-100) on a HaCaT cell line.

The microscopic images have shown clearly that there was no decrease of confluent monolayer, which was treated with negative control (Figure 8a) and possessed complete destruction with a positive control, as depicted in Figure 8b (1% Trixton-100). Whereas both concentraions of the PHA/Ch-hBN (1.0% hBN wrt PHA) nanocomposite showed no cytotoxic effects against the HaCaT cell lines as shown in Figure 8c. Figure 9 illustrates the comprehensive percent cell cytotoxicity data. 

## 6. Comparison of Antibacterial Efficiencies

The antibacterial performance of the synthesized h-BN doped nanocomposite was compared with similar reported studies as illustrated in Table 2. In this investigation, the first two hours bactericidal activity of hBN was found to be 92% and 97% against *E. coli* K1 and MRSA, respectively, which is comparatively much enhanced as compared with other reports. As is apparent from the comparative data shown in Table 2, the synthesized hBN doped nanocomposite offers enhanced antibacterial efficiency and can thus be applicable for efficient in antibacterial treatments and biological applications. 

## 7. Conclusions

An PHA/Ch matrix and hBN nanodiscs-doped nanocomposite scaffolds have been efficaciously designed and fabricated through a facile solvent casting technique. hBN nanodiscs were effectively amalgamated within the inter-cross linked polymeric network of the PHA/Ch. The current examination indicated the synergistic consequence of the thermally stable hBN and the biopolymers, thus leading to the augmentation of the antibacterial activity of the nanocomposite against MDR *E. coli* K1 and MRSA. The fabricated polymeric scaffolds have exhibited superior bactericidal activity and killed pathogenic bacteria competently in a small time interval. Besides, nanocomposite has good cytocompatibility with human cells, as there was a minimal cytotoxicity observed. In conclusion, the multifunctional PHA/Ch-hBN nanocomposites have exhibited broad spectrum antibacterial efficiency and cytocompatibility within appropriate concentrations. Therefore, the synthesized nanocomposites can be considered as safe, efficient, and stable antibacterial scaffolds for biological applications.

## Figures and Tables

**Table 1:** PHA/Ch and hBN-doped PHA/Ch nanocomposite scaffolds antibacterial activity against *E. coli* K1and MRSA.

Test Samples	Antibacterial Activity against *E. coli* K1	Antibacterial Activity against MRSA
PHA/Ch	-	-
PHA/Ch-hBN (0.1 wt%)	+	+
PHA/Ch-hBN (0.5 wt%)	+	+
PHA/Ch-hBN (1 wt%)	+	+
Gentamicin	+	+

**Table 2:** Comparative study of the antibacterial efficiencies of the different composites.

Nano Composites	Conc.	% Reduction		Time (h)	Proposed Applications	Ref.
		*E.coli*	MRSA			
BN/Ag	70 mg/L	100	-	3	Eternal catalyst and antibacterial	[39]
Gr-Pln	5 mg/mL	-	92	-	Antibacterial applications	[40]
BNAg/TiO_2_	2 mg/mL	100	-	3	Photodegradation and antibacterial applications	[41]
Cu-Go/hBN	-	100	-	24	Biology and medical applications	[42]
PEI/BNNT	1 mg/mL	95	90	2	Nano vector for targeted drug delivery system	[43]
PHA/GAg	-	82	60	2	Antibacterial and sanitization application	[26]
PHA/Ch-hBN	1 mg/mL	92	97	2	Antibacterial and biological applications	This study
